# Lessons from the USAID Child Blindness Programme

**Published:** 2017-08-07

**Authors:** Victoria Sheffield, Priya Adhisesha Reddy

**Affiliations:** 1 President & CEO: International Eye Foundation, Metropolitan Washington DC, USA.; 2 Project Manager: Aravind Eye Hospital and Postgraduate Institute of Ophthalmology, Pondicherry, India and Fulbright Scholar, Hubert H Humphrey Fellow 2016–17, Rollins School of Public Health, Emory University, Atlanta, GA, USA.

**Helping all children who need spectacles means doing more than visiting schools and offering good refractive services. Partnerships with local government, hospitals and community groups are needed, alongside a thorough awareness of gender disparities and disability.**

**Figure:**
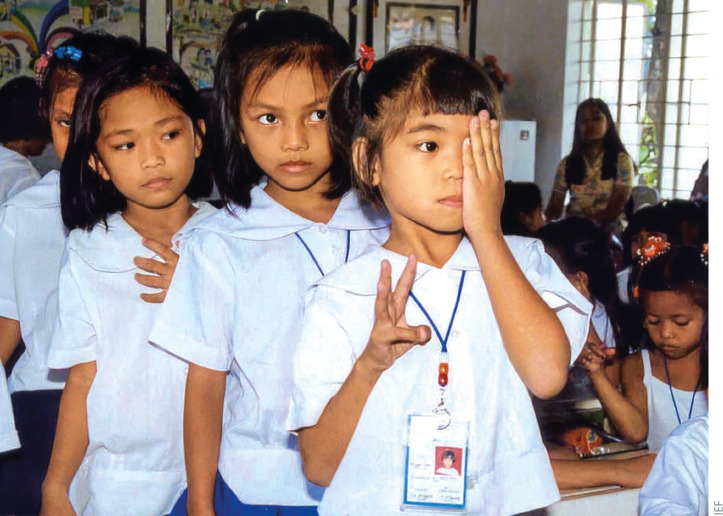
Primary school girls in the Philippines have their vision tested to check whether they need spectacles. PHILIPPINES

The United States Agency for International Development (USAID)'s Child Blindness Program (CBP) focuses on ending avoidable blindness in children. CBP's work includes the provision of sight-restoring surgery, screening children for eye diseases and conditions, and delivering spectacles to school children. Children who are irreversibly blind receive specialised education to learn Braille, use a cane, and improve their daily living skills.

The CBP has identified uncorrected refractive error in children as one of its priorities and has recently funded twelve school eye health projects. In these projects, children were screened at school to identify vision problems; they were then referred for refraction and spectacles, clinical treatment, and/or low vision care, as needed.

At the first CBP regional partners' meeting, held last year, partners from around the world had an opportunity to share field experiences, good practices and lessons learned. The uncorrected refractive error round-table discussion included managers from six of the twelve projects involved in school eye health; their projects were based in Ethiopia, Vietnam, China, Bangladesh and India (two projects). The managers identified the following key issues as having the greatest impact on project success:

- Availability and cost of spectacles
- Income generation
- The quality and availability of eye care professionals
- Case finding, gender disparities and disability
- Follow-up and compliance.

Each of these are discussed in more detail here.

## Availability and cost of spectacles

Since India and many other countries in Asia produce their own spectacles, the projects outside of Africa had the easiest access to spectacles. The project in Ethiopia, however, had ongoing issues with importation, taxation and so on, leading to higher costs and longer lag times between order and delivery. The managers agreed that, ideally, children should not have to wait for more than two weeks before receiving their spectacles. This is because children's visual acuity changes more rapidly than adults' and delays in delivery could result in children receiving spectacles that no longer correct their refractive error.

The cost of plastic lenses was also an issue. For safety reasons, plastic lenses, not glass lenses, should always be used in children's spectacles. However, plastic lenses are more expensive. Two of the six projects represented in the working group were consistently providing plastic lenses. Before receiving funding from the CBP, four projects had used glass lenses when they had insufficient funds to purchase plastic ones.

## Income generation

Income generation through the sale of spectacles was a point of much discussion. The aim is to make a profit on the sale of more expensive spectacle frames in order to cross-subsidise free spectacles for poorer families.

In China, Vietnam and Bangladesh, government hospitals were not always permitted to sell spectacles. To get around this, the project in Vietnam had convinced the government to allow them to sell spectacles in three vision centres (clinics) instead. However, there were doubts about long-term sustainability because there was no certainty that the government would continue to allow the clinics to generate income in this way.

**Figure:**
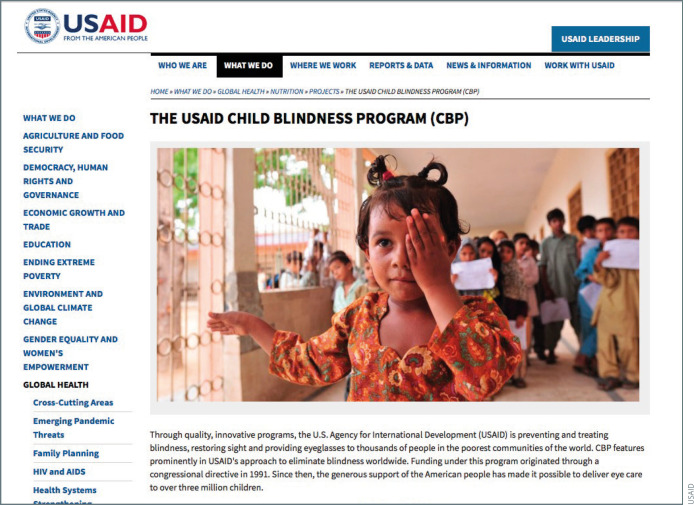
Innovative programs supported by USAID to strengthen eye care services for children.

The CBP-funded project in China used a combined model. In this model, the government provided spectacles at no cost, whereas the private sector sold spectacles of varying styles and prices. People could decide which service to use, depending on their available funds.

## The quality and availability of eye care professionals

There was a general concern about the lack of skilled refraction personnel. The managers from China, Vietnam, and Bangladesh were also concerned about the quality of existing services.

**“India has an education system that promotes the development of eye care professionals (including optometrists) in an efficient and cost-effective manner, supported by strong supervision.”**

The project in Vietnam found that the quality of refraction provided by optometrists was lower than expected. This could be due to inadequate training or perhaps reduced motivation in areas where the profits from spectacle sales were smaller. Vietnam reported a lack of equipment such as Snellen charts, trial lens sets and retinoscopes in district-level government hospitals.

Only the projects in India felt that their refraction services were of acceptable quality. India has an education system that promotes the development of eye care professionals (including optometrists) in an efficient and cost-effective manner, supported by strong supervision.

## Case finding, gender disparities and disability

Most projects in the working group used teachers to conduct vision screening among children aged five years and older. Pathologies such as paediatric cataract, conjunctivitis, strabismus and trauma were also detected. Some projects screened patients with multiple disabilities, who were often identified in specialised schools.

Gender disparities existed in case finding and other activities across all settings. Project staff expressed a desire to address this proactively, but methods such as counselling demanded additional resources and were often not accepted by decision makers, including government officials (who did not always believe the data regarding gender disparity in health outcomes). To counter this, teachers and other health workers involved in CBP projects were taught how gender determines the likelihood that a child will receive services, and how the lack of attention given to girls can lead to poor outcomes later on. CBP projects were also required to disaggregate their data by gender. As a result, project personnel were especially motivated to find, examine and ensure spectacle distribution to girls. This led to greater efforts and resources being applied to gender equity.

Identification of children in blind schools has unique barriers. Families of children in blind schools often do not want to learn that their child has the ability to see, because these schools may have superior facilities in comparison to non-specialised schools. Many are boarding schools where food and clothing are provided. Additionally, parents may receive government stipends if they are caring for a disabled child; they would be reluctant to lose those benefits. Organisations need to make a greater effort to identify children in these schools and to give families incentives to accept care when the child's vision can be recovered.

## Follow-up and compliance

The increased use of cell phones (mobile phones) in the global population is having a positive impact on follow-up and compliance, as project staff are able to reach parents more easily. The Bangladesh project reported using cell phones in 20–25% of their case follow-up work.

Project staff also reported that their organisations are emphasising patient counselling and support, such as transportation costs, in addition to referring children who failed vision screening.

In some instances, teachers were trained to educate families and children regarding the importance of attending follow-up visits and wearing their spectacles. Teachers reinforced this in their classrooms by asking the child if they had received their spectacles and by monitoring whether children wore them. This training, and the fact that teachers are seen as role models, helped to improve acceptance of spectacles.

The managers all agreed that follow-up visits to teachers, after school screening, is best when done more than once after the initial activity.

All the partners maintained records of patient follow-up after school screening.

## Discussion

The topics above highlight the challenges of getting spectacles to the millions of children who need them. Screening is relatively easy and many groups show success finding children who need spectacles. Getting the spectacles to them is far more difficult, however. In some parts of the world, particularly in Asia, frames and blank lenses are increasingly being manufactured in the region at a low cost and with timely shipping. However, in the majority of low- and middle-income countries, frames and lenses are not readily available and must be imported, requiring import tariffs, intermediary companies and high shipping costs. These barriers increase the cost of providing spectacles, leading to high retail prices for parents.

Human resources are a common problem, mentioned across most of the working group participants. Low- and middle-income countries lack sufficient, qualified optometrists and/or refractionists. Ophthalmologists prioritise clinical and surgical care and have little time to perform refraction. Even when there are more personnel available to refract children, the majority are concentrated in urban areas - which means that children in rural areas may not receive the services they need. The quality of training is often poor, leading to incorrect refraction; this can result in non-compliance with spectacle wear or worsening of the child's vision.

**“Schools are an easy target for screening, but very young children, and children with extremely poor vision, do not go to school.”**

Whereas screening is relatively easy, there are barriers to achieving good results. Finding all children who need vision correction can be difficult. Schools are an easy target for screening, but very young children, and children with extremely poor vision, do not go to school. A lot of time and resources are needed in order to develop relationships with community leaders and health professionals in order to find and help all families whose children need eye care.

Follow-up is always challenging, given that many children and their families live in remote areas and some do not have access to a telephone (although that is changing due to the proliferation of cell phones/mobile phones). Therefore, projects should carefully consider how they will ensure that children are properly followed up and also carry on wearing their spectacles.

Cultural prohibitions, bullying in school, incorrect refraction, and financial constraints are among the challenges in getting children to wear their spectacles. Promoting and celebrating spectacle wear can help to improve matters.

In summary, a successful, sustainable URE programme, which ensures that children who need spectacles receive them, must include screening, qualified human resources, training, quality improvement, procurement, monitoring, and financing.

**The challenges of getting government support: an example from India**

Representatives from Aravind Eye Hospitals in India discussed the impact of government involvement (or the lack thereof) on their projects. An example was shared regarding two school screening projects in two adjoining districts (these projects were not funded by USAID's Child Blindness Project, but were given to illustrate an important field reality).

In each case, the local health ministry was asked to commit funds to match the non-governmental organisation's (NGO) contribution of human resources. In one of the districts, the local health ministry was receptive; it provided funds and advertised the screening programme in all local schools. This led to almost 100% of all children being screened, and further government support for annual screening. However, the health ministry in the nearby district declined to work with the same NGO to set up a screening programme. Despite similar geographic conditions and government structures, and the same level of effort from the NGO, the outcome was negative.

These cases show that unique factors such as motivated government staff and representatives can dictate success or failure, despite the efforts of the same NGO in both instances.

This article is made possible by the generous support of the American people through the United States Agency for International Development (USAID) under the terms of contract are the responsibility of the authors and AID-OAA-C-13-00088. The contents do not necessarily reflect the views of USAID or the United States Government.

